# Differences in handgrip strength protocols to identify sarcopenia and frailty - a systematic review

**DOI:** 10.1186/s12877-017-0625-y

**Published:** 2017-10-16

**Authors:** A. R. Sousa-Santos, T. F. Amaral

**Affiliations:** Faculdade de Ciências da Nutrição e Alimentação, Universidade do Porto, Rua Dr. Roberto Frias, 4200-465 Porto, Portugal

**Keywords:** Sarcopenia, Frailty, Handgrip strength, Older adults

## Abstract

**Background:**

Hand grip strength (HGS) is used for the diagnosis of sarcopenia and frailty. Several factors have been shown to influence HGS values during measurement. Therefore, variations in the protocols used to assess HGS, as part of the diagnosis of sarcopenia and frailty, may lead to the identification of different individuals with low HGS, introducing bias. The aim of this systematic review is to gather all the relevant studies that measured HGS to diagnose sarcopenia and frailty and to identify the differences between the protocols used.

**Methods:**

A systematic review was carried out following the recommendations of The Preferred Reporting Items for Systematic Reviews and Meta-Analyses (PRISMA) Statement. PubMed and Web of Science were systematically searched, until August 16, 2016. The evidence regarding HGS measurement protocols used to diagnose sarcopenia and frailty was summarised and the most recent protocols regarding the procedure were compared.

**Results:**

From the described search 4393 articles were identified. Seventy-two studies were included in this systematic review, in which 37 referred to sarcopenia articles, 33 to frailty and two evaluated both conditions. Most studies presented limited information regarding the protocols used.

**Conclusions:**

The majority of the studies included did not describe a complete procedure of HGS measurement. The high heterogeneity between the protocols used, in sarcopenia and frailty studies, create an enormous difficulty in drawing comparative conclusions among them.

## Background

Ageing is accompanied by numerous underlying physiological changes and increasing risk of certain health conditions, such as chronic diseases. These changes that constitute and influence ageing are complex [1]. Sarcopenia and frailty are two geriatric syndromes that are frequently confounded [2].

Sarcopenia was initially proposed by Irwin Rosenberg, in 1989, to define the age-related decrease of muscle mass. It derives from the Greek words ‘sarx’, that means flesh, and ‘penia’, that means loss [3]. In 2009, the International Working Group on Sarcopenia (IWGS) provided a consensus definition describing sarcopenia as the age-associated loss of skeletal muscle mass and function. It was proposed that older patients who presented decline in physical function, strength or overall health should be considered for sarcopenia diagnosis [4]. In 2010, the European Working Group on Sarcopenia in Older People (EWGSOP) released a clinic definition and a consensus diagnostic criteria for age-related sarcopenia. They presented sarcopenia as a syndrome characterised by progressive and generalised loss of skeletal muscle mass and strength with a risk of adverse outcomes such as physical disability, poor quality of life, and death. The diagnosis should consider the presence of low muscle mass and low muscle function (strength or performance) to define conceptual stages as ‘presarcopenia’, ‘sarcopenia’ and ‘severe sarcopenia’ [2].

Frailty is a clinically recognisable state of increased vulnerability resulting from age-associated decline in reserve and function across multiple physiologic systems [5], which is associated with adverse outcomes, such as falls, functional decline, hospitalisations and mortality [6–9]. Even though, there is no single generally accepted clinical definition of frailty, in the Cardiovascular Health Study (CHS) it was defined as a clinical syndrome in which three or more of the following characteristics were present: unintended weight loss, exhaustion, weakness, slow gait speed and low physical activity [10]. Fried’s frailty scale has been the most extensively tested for its validity and is the most widely used instrument in frailty research [11].

Hand grip strength (HGS) is used to diagnose both sarcopenia and frailty [2, 4, 10]. It can be quantified by measuring the amount of static force that the hand can squeeze around a dynamometer [12] and it is an indicator of overall muscle strength [13]. Age and gender are described as the strongest factors influencing HGS in healthy subjects, HGS declines with increasing age [14] and presents lower values for women [15, 16]. It has good intra- and inter-tester reliability and can be recommended the use in clinical practice [17, 18]. HGS can independently identify changes in nutritional status [19]; it responds earlier than anthropometrical measurements to nutritional deprivation and has shown to be significantly associated with sarcopenia [2] and frailty [10].

While HGS is considered a reliable measure to assess muscle strength, several factors have been shown to influence HGS values during measurement. It was reported that a different posture [20], different positions of the elbow [20] and wrist [21], the hand used to test [22] and the setting of the dynamometer [23] may affect the values of strength. It is even reinforced that certain positions can optimise the measurement and produce a maximal HGS. Therefore, variations in the protocols used to assess HGS, as part of the diagnosis of sarcopenia and frailty, may lead to the identification of different individuals with low HGS, introducing bias. This can occur even when the same cut-off points are adopted, which consequently can lead to differences in the number of individuals identified with sarcopenia and frailty.The American Society of Hand Therapists (ASHT) recommended, in 1981, that HGS should be measured with the individuals seated with their shoulders adducted, their elbows flexed 90° and their forearms in neutral position using the Jamar dynamometer [24]. This protocol has been updated with more details of the procedure in 1992 [25], and later in 2015 [26]. In 2011, a new protocol was proposed, the Southampton protocol [27], representing another step towards an improvement of the description of HGS measurement. Nevertheless, there is still a lack of consistency in the studies’ protocols to evaluate HGS used over time.

This systematic review resulted from the need to evaluate the differences between the protocols used for the HGS measurement to diagnose sarcopenia and frailty in older adults. For this reason, this revision represents a step forward towards the standardisation of the procedure. Therefore, the aim of this article is to gather all the relevant studies that measure HGS and to identify the differences between the protocols used. To this end, the proposed systematic review will answer the following questions:

1. Which dynamometer was used for measuring HGS?
2. Which hand was used?
3. What was the individual’s posture?
4. What was the arm position?
5. Which handle position was used?
6. How long did the HGS measurement take?
7. How long were the intervals between the measurements?

## Methods

A systematic review was carried out following the recommendations for reporting systematic reviews and meta-analyses of the Preferred Reporting Items for Systematic Reviews and Meta-Analyses (The PRISMA Statement) [28]. PubMed and Web of Science were systematically searched until August 16, 2016, with no restriction on the year of publication. The search was limited to English, Portuguese, Spanish and French publications and to human subjects. The reference lists within the articles were scanned for any additional references missing from the databases’ search. The following search terms were used: [1] ((hand OR handgrip OR grip OR grasp) AND (force OR strength)) AND (sarcopenia OR frail elderly OR frail OR frailty). Subsequently, search results were inserted in EndNote X7 and duplicates were excluded. All the titles and abstracts were screened based on the eligibility criteria and classified as “relevant” or “not relevant”. Full texts of eligible articles were assessed and read. Those that met all criteria were included.

### Eligibility criteria

Studies were included if [1] participants were aged 65 years or older within well-defined samples, with a clear description of the inclusion and exclusion criteria; [2] sarcopenia and frailty were considered as outcomes, in which HGS was used to identify this condition; [3] a description of the protocol used to measure handgrip strength was provided; [4] the outcome measures described are: type of dynamometer for the assessment of HGS, individual’s position (including shoulder, elbow, arm and handle position and posture), hand dominance, number of repetitions, acquisition and rest time, encouragement and handgrip strength values.

Randomised control trials, cohort studies, case control studies and cross-sectional studies were included, and meta-analyses or review articles, case reports, case series, meetings’ proceedings, conference summaries and duplicate records were excluded. Articles were not included if information about either the posture of the individual, or concerning the arm position (shoulder, elbow or wrist) was absent. When the complete procedure was not described but a reference was made to another article, we searched for the missing parts of the procedure. If the article did not add more details regarding the procedure, it was still excluded. In case of disagreement about the inclusion of a study, the reviewers discussed their opinions to reach consensus. The studies were divided into two subgroups: [1] articles about sarcopenia and [2] articles about frailty. Final studies selected for inclusion in each category were independently compiled in data tables. Articles which presented the same data as an earlier study were still excluded.

## Results

**Fig. 1:**
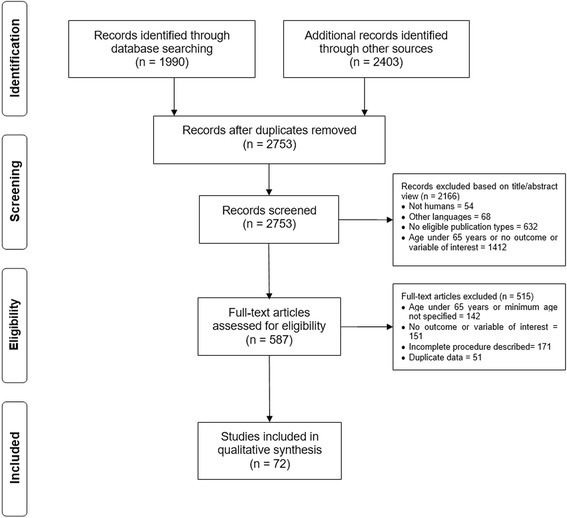
Flow diagram of the literature search and selection process

From the described search 4393 articles were identified. After removing duplicates, a total of 2753 articles remained. From these, after screening for title and abstract 2166 articles were excluded. Five hundred and eighty-seven full-text articles were assessed for eligibility and 515 references were excluded. Seventy-two studies were found eligible and, therefore, included in this systematic review. Figure 1 presents a flow diagram of the literature search and of the selection process.

The studies comprised in this systematic review were published between 2003 and 2016. Fifty-two were cross-sectional studies, 17 were cohorts, and three were clinical trials. The sample size ranged between 24 and 11,844 individuals.

From the articles included, 37 studies referred to sarcopenia, 33 to frailty and two evaluated both conditions. The EWGSOP and the CHS definitions were used in the majority of studies to diagnose sarcopenia and frailty.

### Description of HGS measurement

Most studies presented limited information regarding the protocols used. As shown in both Tables 1 and 2, all 72 studies described the dynamometer used, but only five specified if it was calibrated for the study. Although, there was a wide range of equipment used, the Jamar dynamometer was the most mentioned (*n* = 35), followed by the Smedley dynamometer (*n* = 10). Sixty-six studies described the posture of the individual, in which the majority was measured in a sitting position (*n* = 47), and 19 were in a standing position. Three studies mentioned variations regarding the posture, depending on the ability of the individuals.

**Table 1:** Details and HGS protocols of the studies that diagnose sarcopenia, included in this systematic review

Study details	Author	Sample	Size	Age	Dynamometer	Repetitions	Hand	Posture	Shoulder position	Elbow position	Wrist position	Handle position	Encouragement	Acquisition time	Rest time	HGS analysis	Cut-off values
Cross-sectional study Toulouse and Lyon, France	Abellan van Kan et al. [52]	Community-dwelling older women from the EPIDOS cohort	3025	≥75	Martin vigorimeter, Medizin Tecnik, Tuttlingen, Germany	3	Dominant	Standing upright	Adducted	180°	–	Adjusted to a comfortable position	–	–	–	Higher value	Lowest 25%
Cross-sectional study Turkey	Akin et al. [53]	Community-dwelling older adults from KEHES Study	879	≥60	Takei TKK 5401 digital handgrip dynamometer, Takei, Niigata-City, Japan	3	Dominant	Standing upright	Adducted	90°	–	–	–	–	–	Higher value	Fried’s criteria^*^
Cross-sectional study S. Paulo, Brazil	Alexandre Tda et al. [54]	Older urban population from the SABE Study	1149	≥60	Takei Kiki Kogyo TK 1201, Tokyo, Japan	2	Dominant	Sitting position	–	Resting on the table (forearms too)	Palms facing up	Adjusted to a comfortable position	–	–	1 min	Higher value	M: <30 kgf W: <20 kgf
Cross-sectional study Milan, Italy	Barichella et al. [55]	Consecutive patients from a specialised tertiary care center	364	≥65	DynEx digital hand dynamometer, Akern/MD Systems, Florence, Italy	3	Dominant	Sitting position	Adducted and neutrally rotated	90° Forearm neutral	Neutral	–	–	–	–	Mean value	M: <30 kgf W: <20 kgf
Cross-sectional study The Netherlands	Bastiaanse et al. [56] ^(a)^	Adults with intellectual disabilities from the HA-ID study	884	≥50	Jamar hand dynamometer, Sammons Preston Rolyan, USA	6	Both	Sitting position	Adducted and neutrally rotated	90° Forearm neutral	Neutral	2nd	–	–	1 min	Higher value	M: <30 kgf W: <20 kgf
Cross-sectional study Liège, Belgium	Beaudart et al. [57] ^(d)^	Consecutive outpatients from an osteoporotic and geriatric department of a clinic and community-dwelling older adults	250	≥65	Hydraulic and pneumatic dynamometer Saehan Corporation, MSD Europe, Bvba, Belgium (calibrated)	6	Both	Sitting position	–	Forearms resting on the arms of the chair	Neutral position, over the end of the arm of the chair, thumb facing upwards	Adjusted so that the thumb is round one side of the handle and the four fingers are around the other side	Yes	–	–	Higher value	M: <30 kgf W: <20 kgf
Cross-sectional study Liège, Belgium	Beaudart et al. [58] ^(d)^	Community-dwelling older adults from the SarcoPhAge study	534	≥65	Hydraulic dynamometer Saehan Corporation, MSD Europe, Bvba, Belgium (calibrated)	6	Both	Sitting position	–	Forearms resting on the arms of the chair	Neutral position, over the end of the arm of the chair, thumb facing upwards	Adjusted so that the thumb is round one side of the handle and the four fingers are around the other side	Yes	–	–	Higher value	M: <30 kgf W: <20 kgf
Cross-sectional study The Netherlands	Bijlsma et al. [59]	Young and healthy older Europeans from the Leiden Longevity Study	654	38–82	Jamar hand dynamometer, Sammons Preston Inc., Bolingbrook, IL, USA	3	Dominant	Standing upright	Abducted	180°	–	Adjusted to hand size (middle phalanx rested on the inner handle)	–	–	–	Higher value	M: <30.3 kgf W: <19.3 kgf
Cross-sectional study Leiden, The Netherlands; Jyvaskyla, Finland; Tartu, Estonia; Paris, France and Manchester, United Kingdom (UK)	Bijlsma et al. [60]	Middle to older participants from the MYOAGE study	452	18–30/ 69–81	Jamar hand dynamometer, Sammons Preston, Inc., Bolingbrook, IL, USA	6	Both	Standing upright	Abducted	180°	–	Adjusted to hand size	–	–	–	Higher value	**
Cross-sectional study Guelph, Ontario, Canada	Campbell et al. [61]	Assisted-living older adults	40	≥65	Vernier digital hand dynamometer and collected using LoggerPro software, Vernier, OR, USA; 60 Hz	6	Both	Sitting position	Adducted	90°	Dynamometer vertical	–	Yes	Self-selected pace	–	Higher value	M: <30 kgf W: <20 kgf
Prospective cohort study Northern Italy	Cerri et al. [62]	Consecutively admitted older inpatients of an Acute Geriatric Clinic, S. Gerardo University Hospital	103	≥65	Jamar hand dynamometer	3	Dominant	Sitting position	Adducted	90° Forearm neutral	Between 0 and 30° extension	–	–	–	1 min	Higher value	M: <30 kgf W: <20 kgf
Cross-sectional study Madrid and Barcelona, Spain	Cuesta et al. [63] ^(a)^	Geriatric outpatients from the ELLI study	298	≥70	Jamar hand dynamometer	3	Dominant	Sitting position	Adducted and neutrally rotated	90° Forearm neutral	Neutral	2nd	–	–	1 min	Higher value	M: <30 kgf W: <20 kgf
Cross-sectional study Midwestern United States of America (USA)	Fukuda et al. [64]	Caucasian ambulatory individuals	107	65–89	DHS-176 digital handgrip dynamometer, Detecto, Webb City, MO	3	Dominant	Standing upright	Adducted	90°	–	–	–	3 to 5 s	–	Mean value	**
Cross-sectional study Spain	Garatachea et al. [65]	Caucasian community-dwelling older adults from two geriatric nursing homes	81	71–93	Smedley digital hand dynamometer, Sportstek,VIC, Australia	3	Non-dominant	Standing upright	Abducted	180°	–	Adjusted to hand size	–	–	30 to 60 s	Higher value	**
Prospective cohort study Spain	Gonzalez-Montalvo et al. [66]	Consecutive patients hospitalised for hip fracture in a public 1300-bed university hospital	509	≥65	Jamar hydraulic dynamometer, Sammons Preston, Bolingbrook, IL, USA	3	Dominant	Sitting position	–	Forearms resting on the arms of the chair	Neutral, over the end of the arm of the chair, thumb facing upwards	Adjusted so that the thumb is round one side of the handle and the four fingers are around the other side	Yes	–	–	Higher value	M: <30 kgf W: <20 kgf
Cross-sectional study USA	Gray et al. [67]	Community-dwelling older adults	43	≥65	Takei Scientific Instruments digital grip strength dynamometer, Niigata City, Japan	3	Preferred hand	Standing upright	–	Arms down by the side	Neutral	Interphalangeal joint of the index finger maintained at 90°	Yes	Minimum of 3 s	1 min	Higher value	**
Cross-sectional study Taipei, Taiwan	Han et al. [68]	Healthy volunteers from the Taiwan Fitness for Seniors Study	878	≥65	Baseline hydraulic dynamometer, Fabrication Enterprises Inc., Irvington, NY, USA	3	Dominant	–	Adducted	90° Forearm neutral	–	–	–	–	–	Higher value	M: <30 kgf W: <20 kgf
Cross-sectional study 6th district of Tehran, Iran	Hashemi et al. [69] ^(c)^	Community-dwelling individuals from the SARIR study	300	≥55	Baseline pneumatic squeeze bulb dynamometer, Jamar, Inc. USA: c7489–02 Rolyan (calibrated)	6	Both	Sitting position	Adducted and neutrally rotated	90° Forearm neutral	Neutral	2nd	–	–	30 s	Mean value	Compared with normative data from Merkies et al. [70]
Cross-sectional study Northern Bavaria, Germany	Kemmler et al. [71]	Community-dwelling German women from the FORMoSA study	1325	≥70	Jamar hand dynamometer, Sammons Preston Inc., Bollington, USA	2	Both	Standing upright	–	Arms down by the side	–	Adjusted to hand size	–	–	–	Higher value	W: <20 kgf
Prospective cohort study I-Lan County, Taiwan	Lee et al. [72]	Young healthy volunteers and older adults from the I-Lan Longitudinal Ageing Study	508	20–40/ ≥65	Smedley hand dynamometer, TTM, Tokyo, Japan	3	Dominant	Standing upright	Abducted	180°	–	–	–	–	–	Higher value	M: <22.4 kgf W: <14.3 kgf
Cross-sectional study Korea	Lee et al. [73] ^(b)^	Ambulatory women from the University Hospital Menopause Clinic	196	≥65	Jamar hand dynamometer, Sammons Preston Inc., Bolingbrook, IL, USA	3	Dominant	Sitting position	Adducted and neutrally rotated	90° Forearm neutral	Between 0 and 30° dorsiflexion	2nd	–	–	–	Mean value	W: <18 kgf
Cross-sectional study Tamana, Japan	Maeda et al. [74]	Patients admitted to acute phase wards from Tamana Regional Health Medical Center	224	≥65	Smedley hand dynamometer, TTM, Tokyo, Japan	2	Dominant	Standing or sitting position, depending on their ability	–	–	–	–	–	–	–	Higher value	M: <26 kgf W: <18 kgf
Cross-sectional study Salvador, Bahia, Brazil	Martinez et al. [75]	Hospitalised elderly patients in a multi-specialty hospital	110	≥60	Saehan hydraulic dynamometer, Saehan Corporation, 973, Yangdeok-Dong, Masan 630–728, Korea	3	–	Sitting position	–	90°	–	–	–	–	1 min	Higher value	M: <30 kgf W: <20 kgf
Cross-sectional study Guelph, Canada	McIntosh et al. [76]	Community-dwelling older adults	85	≥65	Vernier digital hand dynamometer and collected using LoggerPro software, Vernier, OR, USA; 60 Hz	6	Both	Standing upright	Adducted	90°	–	–	Yes	–	–	Higher value	M: <30 kgf W: <20 kgf
Prospective cohort study Reykjavik, Iceland	Mijnarends et al. [77]	Community-dwelling older adults from the AGES-Reykjavik Study	2309	66–93	Good Strength software, Metitur, Finland	3	Dominant	Sitting position	Relaxed	90°, neutral	Attached by belts to a strain-gauge system, thumb up	–	Yes	4–5 s	30 s	–	M: <30 kgf W: <20 kgf
Prospective cohort study Seongnam, Korea	Moon et al. [78]	Community-dwelling older adults from the Korean Longitudinal Study on Health and Aging	297	≥65	Jamar hydraulic hand dynamometer, Sammons Preston, Bolingbrook, IL, USA	2	Dominant	Sitting position	Adducted	90° Forearm neutral	–	Adjusted to a comfortable position	–	–	1 min	Mean value	M: <26 kgf W: <16 kgf
Cross-sectional study London, Ontario, Canada	Morat et al. [79]	Healthy and independent living older adults from the Canadian Centre for Activity and Aging	24	≥65	Smedley hand dynamometer, TTM, Tokyo, 100 kg	6	Both	Standing upright	–	90° Forearm neutral	Neutral	–	–	–		Higher value	M: <30 kgf W: <20 kgf
Cross-sectional study Goiâna, Brazil	Pagotto et al. [80] ^(b)^	Community-dwelling older adults	132	≥60	CROWN hydraulic dynamometer	2	Dominant	Sitting position	Adducted and neutrally rotated	90°	Extended between 0 and 30° dorsiflexion	2nd	–	6 s	1 min	Both values	M: <30 kgf W: <20 kgf and Fried’s criteria^*^
Cross-sectional study UK	Patel et al. [81] ^(d)^	Community-dwelling older adults from the Hertfordshire Sarcopenia Study	1890	68–77	Jamar hand dynamometer	6	Both	Sitting position	–	Forearms resting on the arms of the chair	Neutral, over the end of the arm of the chair, thumb facing upwards	Adjusted so that the thumb is round one side of the handle and the four fingers are around the other side	Yes	–	–	Higher value	M: <30 kgf W: <20 kgf
Cross-sectional study Pavia, Italy	Rondanelli et al. [82]	Older adults consecutively admitted to a physical medicine and rehabilitation division, in Santa Margherita institute	159	≥65	Jamar 5030 J1 hydraulic hand dynamometer, Sammons Preston Rolyan, Bolingbrook, IL,USA	4	–	Sitting position	–	Comfortable arm position	–	–	Yes	5 s	1 min	Mean value of the last three efforts	**
Prospective cohort study Barcelona, Spain	Sanchez-Rodriguez et al. [83] ^(d)^	Consecutive hospitalised patients from a postacute care geriatric unit	100	≥70	Jamar hand dynamometer, Nottinghamshire, UK	3	–	Sitting position	–	Forearms resting on the arms of the chair	Neutral, over the end of the arm of the chair, thumb facing upwards	Adjusted so that the thumb is round one side of the handle and the four fingers are around the other side	Yes	–	–	Higher value	Compared with normative data from Luna-Heredia et al. [16]
Retrospective cohort study Kuopio, Eastern Finland	Sjoblom et al. [84]	Finnish postmenopausal women from the OSTPRE study	590	65–72	Pneumatic hand-held dynamometer Martin Vigorimeter, Germany	3	–	Sitting position	–	–	–	–	–	–	–	Mean value	Lowest 25%
Cross-sectional study Porto, Portugal	Sousa et al. [85] ^(b)^	Hospitalised adult patients from medical and surgical wards in a general and teaching hospital	608	≥18	Jamar hydraulic hand dynamometer, Sammons Preston, Bolingbrook, IL, USA (calibrated)	3	Non-dominant	Sitting position	Adducted and neutrally rotated	90°	Between 0 and 30° dorsiflexion	2nd	–	–	1 min	Higher value	M: <30 kgf W: <20 kgf
Cross-sectional study Berlin, Germany	Spira et al. [86]	Community-dwelling older adults from the BASE-II study	1405	60–80	Smedley hand dynamometer, Scandidact, Denmark	6	Both	Standing upright	Adducted and neutrally rotated	90° Forearm neutral	Neutral	–	–	–	–	Higher value	Fried’s criteria^*^
Cross-sectional study Manchester, UK and Leuven, Belgium	Verschuere et al. [87] ^(d)^	Men from the European Male Ageing Study	679	40–79	Jamar hand dynamometer, TEC Inc., Clifton, NJ	6	Both	Sitting position	–	Forearms resting on the arms of the chair	Neutral, over the end of the arm of the chair, thumb facing upwards	Adjusted so that the thumb is round one side of the handle and the four fingers are around the other side	Yes	–	–	Higher value	Fried’s criteria^*^
Multicentre cohort study Italy	Vetrano et al. [88]	Older adults admitted to acute care wards, of seven Italian hospitals, from the CRIME study	770	≥65	North Coast hydraulic hand dynamometer, North Coast Medical Inc., Morgan Hill, CA	4	Both	Sitting position or lying at 30° in bed (when unable to sit)	–	90° or with elbows supported	Neutral	–	–	–	–	Higher value	M: <30 kgf W: <20 kgf
Cohort study Ankara, Turkey	Yalcin et al. [89]	Residents in Seyranbagları Nursing Home and Rehabilitation Center	141	≥65	Takei Scientific Instruments, Niigata, Japan	2	Dominant	–	Abducted (30°)	180°	Palm perpendicular to the shoulder line	–	–	5 s	–	Mean value	M: <30 kgf W: <20 kgf
Cross-sectional study Obu, Aichi, Japan	Yoshida et al. [90]	Community-dwelling older adults from Obu Study of Health Promotion for the Elderly	4811	≥65	Grip-D hand dynamometer, Takei, Niigata, Japan	1	Dominant	Standing upright	–	–	–	–	–	–	–	Single value	M: <28.8 kgf W: <18.2 kgf
Cohort study North west regions and Western suburbs of Adelaide, Australia	Yu et al. [91]	Community-dwelling individuals, from the CASA, FAMAS and NWAHS studies	1123	≥18	Lafayette Instrument Company, IN, USA (CASA and NWAHS), Smedley, Chicago, IL (FAMAS)	3	Dominant	Sitting position	–	Arm supported by a horizontal surface	–	–	–	–	–	Mean value	M: <30 kgf W: <20 kgf

*S* Seconds; *Min* Minutes; *M* Men; *W* Women

^(a)^ Study cited the ASHT 1981 protocol

^(b)^ Study cited the ASHT 1992 protocol

^(c)^ Study cited the ASHT protocol, without specifying which protocol year was used

^(d)^ Study cited the Southampton protocol

^*^ Fried’s criteria (Cut-off points for handgrip strength) Men: ≤29 kgf (BMI ≤ 24 kg/m^2^); ≤30 kgf (BMI 24.1–26 kg/m^2^); ≤30 kgf (BMI 26.1–28 kg/m^2^); ≤32 kgf (BMI > 28 kg/m^2^) / Women: ≤17 kgf (BMI ≤ 23 kg/m^2^); ≤17.3 kgf (BMI 23.1–26 kg/m^2^); ≤18 kgf (BMI 26.1–29 kg/m^2^); ≤21 kgf (BMI > 29 kg/m^2^)

^**^ Not defined due to the type of analysis conducted by the study

**Table 2:** Details and HGS protocols of the studies that diagnose frailty, included in this systematic review

Study details	Author	Sample	Size	Age	Dynamometer	Repetitions	Hand	Posture	Shoulder position	Elbow position	Wrist position	Handle position	Encouragement	Acquisition time	Rest time	HGS analysis	Cut-off values
Multicentric prospective cohort study Burgos, Albacete and Madrid, Spain	Abizanda et al. [92] ^(c)^	Institutionalised older adults, in four nursing homes from the ACTIVNES study	91	≥70	Jamar hand dynamometer, Sammons Preston Rolyan, Bolingbrook, IL	3	–	Sitting position	Adducted and neutrally rotated	90° Forearm neutral	Neutral	2nd	–	–	–	Higher value	Fried’s criteria^*^
Cross-sectional study Alexandria, Egypt	Abou-Raya et al. [93]	Consecutive patients with congestive heart failure	126	≥65	Jamar hand dynamometer	2	Dominant	Sitting position	Adducted	90°	Between 0 and 30° dorsiflexion and 0 and 15° ulnar deviation	2nd	Yes	–	–	–	M: ≤21 kgf W: ≤14 kgf
Cross-sectional study USA	Bandeen-Roche et al. [94]	Older adults from the 2011 baseline of the National Health and Aging Trends Study	7439	≥65	Jamar digital hand dynamometer	2	Dominant	Sitting position	Adducted	90°	Dynamometer or forearm resting on the table	2nd	Yes	–	–	Higher value	Lowest 20% within 8 sex and BMI categories
Cross-sectional study The Netherlands	Bastiaanse et al. [56] ^(a)^	Adults with intellectual disabilities from the HA-ID study	884	≥50	Jamar hand dynamometer, Sammons Preston Rolyan, USA	6	Both	Sitting position	Adducted and neutrally rotated	90° Forearm neutral	Neutral	2nd	–	–	1 min	Higher value	Fried’s criteria^*^
Cross-sectional study Liège, Belgium	Beaudart et al. [58] ^(d)^	Community-dwelling older adults from the SarcoPhAge study	534	≥65	Hydraulic dynamometer Saehan Corporation, MSD Europe, Bvba, Belgium (calibrated)	6	Both	Sitting position	–	Forearms resting on the arms of the chair	Neutral position, over the end of the arm of the chair, thumb facing upwards	Adjusted so that the thumb is round one side of the handle and the four fingers are around the other side	Yes	–	–	Higher value	Fried’s criteria^*^
Cross-sectional study England	Buttery et al. [95]	Consecutively patients from three elderly care wards of an urban teaching hospital	44	67–91	Jamar isometric hand dynamometer, Sammons Preston, Bolingbrook, Illinois, USA	6	Both	Sitting position	Adducted and neutrally rotated	90°	Between 0 and 30° dorsiflexion and 0 and 15° ulnar deviation	2nd	Yes	–	–	Higher value	Compared with normative data from Bohannon et al. [96]
Cross-sectional study Germany	Buttery et al. [97]	Community-dwelling older adults from the DEGS1	1843	65–79	Smedley hand dynamometer, Scandidact, Denmark, 100 kg	4	Both	Standing upright	–	–	–	–	–	–	–	Higher value	Fried’s criteria^*^
Cross-sectional study Urban administrative section of Taipei, Taiwan	Chang et al. [98]	Community-dwelling older adults	234	≥65	Handgrip dynamometer, Fabrication Enterprises, Inc., Irvington, NY	–	Both	–	Adducted	90°	–	–	Yes	–	–	–	Lowest 20% at baseline
Cross-sectional study Saint Bruno,Québec, Canada and Santa Cruz, Rio Grande do Norte, Brazil	Da Camara et al. [99]	Community-dwelling older adults	124	65–74	Jamar hand dynamometer, Jamar, Irvington, NY, USA	3	–	Sitting position	Adducted and neutrally rotated	90° Forearm neutral	Neutral	Adjusted to a comfortable position between the 2nd or 3th handle	–	–	1 min	Mean value	Fried’s criteria^*^
Cross-sectional study Chicago, USA	Danilovich et al. [100] ^(b)^	Convenience sample of older adults	42	≥65	Jamar hand hydraulic dynamometer	4	Both	Sitting position	Adducted and neutrally rotated	90°	Between 0 and 30° dorsiflexion	2nd	–	–	–	Higher value	M: <30 kgf W: <20 kgf
Cross-sectional study Denmark	Dato et al. [101]	Community-dwelling older adults	3719	≥70	Smedley hand dynamometer TTM	3	Dominant	Sitting position	Adducted	–	–	–	–	–	–	Higher value	**
Cross-sectional study The Netherlands	Evenhuis et al. [102]	Individuals with borderline to profound intellectual disabilities of three care provider services from the HA-ID Study	848	≥50	Jamar hand dynamometer, 5030 J1, Sammons Preston Rolyan, Dolgeville, NY	6	Both	Sitting position	Adducted and neutrally rotated	90°	Between 0 and 30° dorsiflexion and 0 and 15° ulnar deviation	2nd	Yes	–	–	–	Fried’s criteria^*^
Prospective cohort study USA	Fried et al. [10]	Community-dwelling older adults from the Cardiovascular Health study	5317	≥65	Jamar hand dynamometer	3	Dominant	Sitting position	–	90°	–	2nd	Yes	–	–	Mean value	Fried’s criteria^*^
Cross-sectional study The Kolpino district, St. Petersburg, Russia	Gurina et al. [103]	Community-dwelling older adults from the “Crystal” Study	611	≥65	Carpal dynamometer (DK-50, Nizhni Tagil, Russian Federation)	6	Both	Standing upright	Arms hanging down at the sides	–	–	–	–	–	30 s	Mean value	Lowest 20%, adjusted for sex and BMI
Cross-sectional study Vienna, Austria.	Haider et al. [104] ^(d)^	Pre-frail and frail community-dwelling older adults	83	≥65	Jamar hydraulic hand dynamometer, Lafayette, Louisiana	6	Both	Sitting position	–	Forearms resting on the arms of the chair	Neutral, over the end of the arm of the chair, thumb facing upwards	Adjusted so that the thumb is round one side of the handle and the four fingers are around the other side	Yes	–	1 min	Higher value	**
Cross-sectional and prospective cohort study The Netherlands	Hoogendijk et al. [105]	Older adults from the Longitudinal Aging Study Amsterdam	1115	≥65	Takei TKK 5001, Takei Scientific Instruments, Tokyo, Japan	4	Both	Standing upright or sitting position when the participant was not able to stand	–	180°	–	–	–	–	–	Sum of the highest values of each hand	Fried’s criteria^*^
Cross-sectional study Seoul, Korea	Kang et al. [106]	Female outpatients from the department of family medicine at Kangbuk Samsung Hospital	121	≥65	Lavisen electronic hand grip dynamometer KS 301, Lavisen Co. Ltd., Namyangju, Korea	–	Right	–	Abducted	180°	–	Medial phalange of the third finger perpendicular to the handle	–	–	–	–	≤14.5 kgf
Cross-sectional study Seoul and Gyeonggi province, Korea	Kim et al. [107]	Older adults who registered at six senior welfare centers	486	≥65	Jamar hydraulic hand dynamometer; Sammons Preston, Bolingbrook, IL, USA	2	–	–	Abducted	180°	–	–	–	–	–	Higher value	Lowest 20%, adjusted for sex and BMI
Cross-sectional study Beaver Dam, Wisconsin	Klein et al. [108]	Adults and older adults from the Beaver Dam Eye Study	2962	≥53	Lafayette hand dynamometer, Model 78,010, Lafayette Instrument Company, Lafayette, Indiana	4	Both	Standing upright	Abducted	180°	–	Adjusted to hand size	–	–	–	Mean value for the dominant hand	M: ≤ 34.5 kgf W: ≤ 18.5 kgf
Randomised controlled trial Itabashi Ward, Tokyo, Japan	Kwon et al. [109]	Pre-frail community-dwelling older women	89	≥70	Smedley hand dynamometer, Yagami, Tokyo, Japan	2	Dominant	Standing upright	Arms hanging naturally at their sides	–	–	–	–	–	–	Higher value	W: ≤23 kgf at baseline
Cohort study Korea	Lee et al. [110]	Community-dwelling older adults from the Living profiles of Older People Survey	11,844	≥65	Tanita, No. 6103, Japan	4	Both	–	Elbow by the side of the body	90°	–	–	–	–	–	Higher value	Lowest 20%, adjusted for sex and BMI
Prospective cohort study Boston, Massachusetts, USA	Mohr et al. [111]	Community-dwelling men from the Massachusetts Male Aging study	646	50–86	Jamar hydraulic hand dynamometer, Sammons Preston, Bolingbrook, IL	2	Dominant	Sitting position	Arms at their sides	90° Forearm neutral	Neutral	Adjusted to hand size	–	3 s	1 min	Higher value	M: ≤28 kgf (BMI ≤ 24.9 kg/m^2^); ≤30 kgf (BMI 25.0–27.2 kg/m^2^); ≤32 kgf (BMI > 27.2 kg/m^2^)
Prospective cohort study Barcelona, Spain	Mora et al. [112]	Community-dwelling women from the Mataró Ageing Study	110	≥70	Jamar hand dynamometer	3	Non-dominant	Sitting position	Adducted and neutrally rotated	90° Forearm neutral	Between 0 and 30° dorsiflexion and between 0 and 15° ulnar deviation	–	Yes	–	–	Mean value	Fried’s criteria^*^
Cross-sectional study Belo Horizonte, Brazil	Moreira et al. [113] ^(b)^	Community-dwelling older women with type 2 diabetes	99	65–89	Jamar hand dynamometer	3	Dominant	Sitting position	Adducted and neutrally rotated	90° Forearm neutral	Between 0 and 30° dorsiflexion	2nd	Yes	–	–	Mean value	Fried’s criteria^*^
Double-blind, randomised, controlled trial Rotterdam, The Netherlands	Muller et al. [114]	Community-dwelling older men	100	≥70	Jamar hand dynamometer, Horsham, PA	3	Non-dominant	Sitting position	Adducted and neutrally rotated	90° Forearm neutral	Between 0 and 30° dorsiflexion and between 0 and 15° ulnar deviation	–	Yes	–	–	Mean value	**
Cross-sectional study Dimantina, Brasil	Parentoni et al. [115] ^(c)^	Convenience sample of older women	106	≥65	Saehan dynamometer, SH5001 (calibrated)	3	Dominant	Sitting position	Adducted and neutrally rotated	90° Forearm neutral	Neutral	2nd	Yes	–	1 min	Mean value	Fried’s criteria^*^
Cross-sectional study Calabria district, Italy	Passarino et al. [116]	Community-dwelling older adults	369	65–85	Smedley hand dynamometer TTM	3	Dominant	Sitting position	Adducted	–	–	–	–	–	–	Higher value	**
Cohort study Texas, New Mexico, Colorado, Arizona and California, USA	Samper-Ternent et al. [117]	Non-institutionalised Mexican Americans from the Hispanic Established Population for the Epidemiological Study of the Elderly	1370	≥65	Jamar hydraulic hand dynamometer, Model 5030 J1, J.A. Preston Corp., Clifton, NJ	2	Dominant	Sitting position	–	Resting on the table	Palm facing up	Adjusted to a comfortable position	Yes	–	–	Higher value	Lowest 20%, adjusted for sex and BMI
Cohort study United States and Denmark	Sanders et al. [118]	Community-dwelling individuals from The Long Life Family Study	4875	32–105	Jamar hydraulic hand Dynamometer, Lafayette, IN	2	Dominant	Sitting position	–	–	–	–	–	–	–	Mean value	Lowest 25%, adjusted for sex and BMI
Cross-sectional study Saarland, Germany	Saum et al. [119] ^(d)^	Community-dwelling adults from ESTHER study	3112	≥59	Jamar hand dynamometer, Lafayette Instrument Company, Lafayette, IN	3	Dominant	Sitting position	–	Forearm resting on the arm of the chair	Neutral, over the end of the arm of the chair, thumb facing upwards	Adjusted so that the thumb is round one side of the handle and the four fingers are around the other side	Yes	–	–	Higher value	M: <30 kgf W: <20 kgf and Fried’s criteria^*^
Cross-sectional study Lausanne, Switzerland	Seematter-Bagnoud et al. [120]	Community-dwelling older adults from the Lc65+ study	861	65–70	Baseline hydraulic dynamometer	3	Right	Sitting position	Adducted and neutrally rotated	90°	Between 0 and 30° dorsiflexion and 0 and 15° ulnar deviation	2nd	Yes	–	–	Higher value	Fried’s criteria^*^
Randomised, Double-Blind, Placebo-Controlled Trial The Netherlands	Tieland et al. [121]	Frail older adults	62	≥65	Jamar hand dynamometer, Jackson, MI, USA	6	Both	Sitting position	–	90°	–	–	–	–	–	–	Fried’s criteria^*^
Cross-sectional study Portugal	Vieira et al. [122] ^(c)^	Institutionalised older adults from three urban residential homes	50	68–99	Jamar hydraulic hand dynamometer, J00105	3	Dominant	Sitting position	Adducted and in extension	90° Forearm neutral	Extended between 0 and 30°	.	–	10 s	1 min	–	M:<30 kgf W: <18 kgf
Cross-sectional study Baltimore, Maryland, USA	Walston et al. [123]	Community-dwelling women from the Women’s Health and Aging Studies I and II	463	70–79	Jamar hand dynamometer, model BK-74978, Fred Sammons, Inc., Burr Ridge, IL	6	Both	Sitting position	Adducted	90°	–	.	Yes	–	–	Higher value of the non-dominant hand	Fried’s criteria^*^
Cross-sectional study Southern Taiwan	Wu et al. [124]	Community-dwelling older adults and outpatients from a hospital-based outpatient clinic	90	≥65	Jamar hand dynamometer, Sammons Preston, Bolingbrook, IL	–	Dominant	Sitting position	–	–	–	–	–	–	–	–	Fried’s criteria^*^

*S* Seconds; *Min* Minutes; *M* Men; *W* Women

^(a)^ Study cited the ASHT 1981 protocol

^(b)^ Study cited the ASHT 1992 protocol

^(c)^ Study cited the ASHT protocol, without specifying which protocol year was used

^(d)^ Study cited the Southampton protocol

^*^ Fried’s criteria (Cut-off points for handgrip strength) Men: ≤29 kgf (BMI ≤ 24 kg/m^2^); ≤30 kgf (BMI 24.1–26 kg/m^2^); ≤30 kgf (BMI 26.1–28 kg/m^2^); ≤32 kgf (BMI > 28 kg/m^2^) / Women: ≤17 kgf (BMI ≤ 23 kg/m^2^); ≤17.3 kgf (BMI 23.1–26 kg/m^2^); ≤18 kgf (BMI 26.1–29 kg/m^2^); ≤21 kgf (BMI > 29 kg/m^2^)

^**^ Not defined due to the type of analysis conducted by the study

Most studies chose to measure HGS only in the dominant hand (*n* = 33), in four studies measurement was obtained from the non-dominant, and in 25 in both dominant and non-dominant. In one study HGS was measured using the preferred hand while the right hand was used in two other studies. In seven articles information about the chosen limb was absent. The position of the shoulder and the elbow was indicated in 46 and 62 studies, respectively, and the wrist position was described in 39 studies. The dynamometer’s handle was referred in 37 articles, while the second handle position was mentioned in 16 articles. Encouragement during the procedure was reported in 26 studies, only nine studies indicated the data acquisition time and, 19 studies specified the rest time. Most studies (*n* = 42) used the higher HGS value for the analysis. The ASHT protocol was mentioned in 11 studies, of which the 1981 protocol was referred twice and the 1992 protocol was cited in five studies. The others did not specify the ASHT protocol used. The Southampton protocol was alluded to in eight studies.

## Discussion

The aim of this systematic review is to identify the HGS protocols used to diagnose sarcopenia and frailty. The heterogeneity in HGS protocols, the wide variability in the criteria used to identify either sarcopenia and frailty and the different inclusion and exclusion criteria in the evaluated studies is an issue in this research field. Indeed, these differences hinder comparison between the studies and hamper progress of the study of these conditions.

We observed that most studies which diagnose these conditions did not mention the protocol used in the measurement of HGS, or did not include a full description of it. Although the ASHT and Roberts et al. proposed standardised protocols, the results of the present review showed high heterogeneity of the chosen procedure. Studies concerning sarcopenia and frailty did not differ in standardised protocols used. Plus, the complete description of the procedure is lacking in most studies. In trying to overcome this problem, some authors raise an additional difficulty when they cite the previous publication of their study protocol.

The parameters regarding the HGS procedure that were presented in the Tables 1 and 2 and its influence in HGS values were evaluated in several studies. As shown below, in spite of some results being similar between the studies, others present contradictory results.

### Dynamometer

The ASHT recommends a calibrated Jamar dynamometer in the second handle position for the measurement of HGS [24–26]. While, the Southampton protocol suggested the handle should be adjusted so that the thumb is round one side of the handle and the four fingers are around the other side and the instrument should feel comfortable in the hand [27].

The Jamar hydraulic dynamometer presents higher intra and inter-individual reliability [17]. Despite this being referred to as the most widely used and tested dynamometer [27], this review shows a great variability in the dynamometers used, regardless of Jamar’s predominance. Present results exhibit a great number of studies which failed to describe if the instruments were properly calibrated for the measurements. A correctly calibrated dynamometer is highly reliable. Nevertheless, it should be recalibrated regularly [29].

Other dynamometers, such as Smedley dynamometer (mechanical) and Martin vigorimeter (pneumatic), measure HGS by a different mechanism [30]. Concerning the Smedley dynamometer, it has shown excellent results regarding its laboratory tested accuracy but, when applied among older adults, it did not produce comparable results to the Jamar hydraulic [31]. Low agreement between Jamar dynamometer and Takei dynamometer was observed [32]. Otherwise, the results of the comparison between the Jamar dynamometer and the Martin vigorimeter in a healthy elderly population, indicate a very high correlation between the two HGS data values [33]. When the hydraulic dynamometers, Baseline and Saehan, were tested they shown to be valid, reliable and comparable to the Jamar dynamometer [34, 35].

### Hand

A summary of the studies comparing HGS in dominant and non-dominant limbs, revealed that it is reasonable to expect greater grip strength in the dominant upper extremity in right-handed individuals [36]. Yet, it is important to consider that the difference between sides varies widely among studied samples and in a significant proportion of individuals the opposite is observed [37, 38].

### Posture and arm position (shoulder, elbow and wrist)

Most studies revised here, a standing or sitting position was selected. In some cases, the position was adapted to the individual’s physical function. The influence of the standing versus sitting posture in HGS values was evaluated and no significant differences were found by several studies [39–41]. When comparing standing versus sitting position, Balogun et al. observed significant differences only between sitting with elbow at 90 degrees and standing with elbow at full extension [20]. These results were in agreement with one study that showed that grip strength is significantly greater when measured with the elbow in the fully extended position [42]. Additionally, even though the posture alone did not significantly influence HGS values, combined with the elbow position it could indicate the presence of an interaction between the elbow position at 180 degrees and a standing position. On the other hand, other results showed a stronger grip strength measurement in the 90 degrees elbow flexed position than in the fully extended position [41, 43].

Su et al. also evaluated different shoulder and elbow positions. They observed that when the shoulder was positioned at 180 degrees of flexion with elbow in full extension the highest mean grip strength measurement was recorded; whereas the position of 90 degrees elbow flexion with shoulder in zero degrees of flexion produced the lowest grip strength score [44]. While, De et al. did not find significant differences when shoulder joints varied between 90 and 180 degrees [41].

Regarding the wrist position, one study suggested that a minimum of 25 degrees of wrist extension was required for optimum grip strength [21]. Later, it was shown that HGS measured with wrist in a neutral position was significantly higher than that in the wrist ulnar deviation [41] and, in another study that the mean grip strength scores were higher for all the tested six positions when wrist was positioned in neutral than in extension position [45].

### Handle position

Some researchers opted for HGS measurement in a standard handle position. However, in others, researchers adapted the handle to hand size or to a comfortable position for the individual. It was suggested that hand size and optimal grip span only correlated in women [46]. Other studies results have shown that the second handle position was the best position for the majority of the participants. Therefore, the authors suggested the use of a standard handle position (second setting) over multiple different positions [23, 47]. This would provide accurate results and increase the comparability of the results [47].

### Repetitions

Mathiowetz et al. suggested that the mean of three trials is a more accurate measure than one trial or even the highest score of three trials [48], while the latter was the most widely adopted by the studies included in this systematic review. In contrast, it was suggested that muscle fatigability might occur with each attempt and one trial is sufficient for the measurement of grip strength [49]. In another study, it was observed that the mean values of grip strength generated for each method of grip strength testing (one trial, the mean of three trials, and the best of three trials) produced comparable results [50].

### Encouragement

To our knowledge, only one research described the effects of the encouragement during HGS measurement. It showed that instruction, verbal encouragement, and visual feedback had critical effects on the handgrip strength and, therefore it should be mentioned in the articles [51]. More than half of the articles included here did not provide a full description of if and how the encouragement was made during the trials.

### Analysis

As described above, most studies used the higher value for the HGS analysis, however other forms of HGS values chosen by the authors, such as the mean or the sum of the values obtained during the measurements was also observed. Hence, the diagnosis of sarcopenia and frailty between the studies is even less comparable.

### Comparison of the protocols

Although the most recent ASHT protocol presents more details regarding the HGS measurement, this protocol has not been adopted by any of the studies included in this revision. Almost every aspect was described in the protocol, making the variations between the studies almost impossible, but also increasing the complexity of the measurement, and therefore the duration of the procedure. Despite the fact that the Southampton protocol referred to all the aforementioned aspects in Table 3, it did not describe in detail the joints position, which could lead to variations in HGS values between the studies.

**Table 3:** Recent HGS protocols proposed

	ASHT protocol – 2015 [26]	Southampton protocol – 2011 [27]
Posture	Subject seated in a chair without arm rests, with feet fully resting on the floor, hips as far back in the chair as possible, and the hips and knees positioned at approximately 90°	Subject seated (same chair for every measurement)
Arm position		Forearms rested on the arms of the chair
-Shoulder	Adducted and neutrally rotated	–
-Elbow	Flexed to 90°, the forearm should be in midprone (neutral)	–
-Wrist	Between 15 and 30° of extension (dorsiflexion) and 0–15° of ulnar deviation	Just over the end of the arm of the chair, in a neutral position, thumb facing upwards
Trials	Three trials	Three trials on each side, alternating sides (start with the right hand)
Dynamometer		
-Model	Jamar dynamometer	Jamar hydraulic hand dynamometer
-Calibration	Yes	–
-Handle position	2nd	Thumb is round one side of the handle and the four fingers are around the other side
Acquisition time	At least 3 s	–
Rest time	At least 15 s	–
Instructions	“This test will tell me your maximum grip strength. When I say go, grip as hard as you can until I say stop. Before each trial, I will ask you ‘Are you ready?’ and then tell you ‘Go’. Stop immediately if you experience any unusual pain or discomfort at any point during testing. Do you have any questions? Are you ready? Go!”. “Harder... harder... harder...Relax”	‘I want you to squeeze as hard as you can for as long as you can until I say stop. Squeeze, squeeze, squeeze, stop’ (when the needle stops rising)
HGS analysis	Mean of three trials	Maximal grip score from all six trials

Due to the great variability in the studies concerning sarcopenia and frailty, namely in the inclusion and exclusion criteria, and in the definition and procedures used to identify these conditions, it is difficult to evaluate the impact of each parameter of the procedure in HGS values. Therefore, to diminish the heterogeneity observed in the studies, the most recent ASHT protocol should be adopted. Variations in the procedure are strongly discouraged, however when it is impossible to fully implement this protocol, namely due to the individuals’ health conditions, any variation should be reported.

### Main topics

The mixed results above discussed reinforce the need to standardise HGS measurement. The difference between the protocols can influence the HGS results and, consequently, affect the comparability between the studies. A common approach would be not only important for research purposes but also for clinical practice. For both sarcopenia and frailty, the major studies that suggested a diagnosis using HGS did not recommend a protocol for its measurement, neither referred to the protocols used to estimate the outlined cut-off points. There is a necessity to include guidelines concerning a standardised protocol in the consensus made by European and International societies. That will allow the results of the studies to be more comparable and more suitable for the application in clinical practice.

In order to describe with precision the handgrip strength protocol used, researchers should always make reference to which protocol was adopted (when applied). For a complete description of the protocol, we suggest that all the points addressed in Table 3 should be mentioned in the methods section of the articles, and therefore include the description of the posture, arm position (including shoulder, elbow and wrist positions), number of trials, characteristics of the dynamometer (brand, model, resolution, calibration and handle position), acquisition and rest time, the applied instructions and the HGS values used in the analysis. The cut-off points to identify low HGS for sarcopenia or frailty should also be stated. Additionally, deviations to the protocol must be described.

## Strengths and limitations

Some strengths of this systematic review can be highlighted. Besides the original search, we additionally handsearched the references of the included articles for a broader research. Plus, for our knowledge there is no other review of literature that comprises a detailed description of the methods of HGS in observational and experimental studies about sarcopenia and frailty in older adults and that considered the most recent protocols proposed for HGS measurement.

This article also had a few limitations. Data was only searched in two databases (Pubmed and Web of Science) and the inclusion of other databases could increase the range of articles found. In addition, we identified three articles in which we could not locate the references made for the full procedure. The focus of the present revision was to gather information regarding HGS methods, hence, we have not evaluated the methodologic quality of the included studies. In our opinion, we do not consider that the limitations would substantially alter our results.

## Conclusion

In conclusion, the majority of the studies included did not describe a complete procedure of HGS measurement. The high heterogeneity between the protocols used, in sarcopenia and frailty related studies, create an enormous difficulty in drawing comparative conclusions among them. Even though, there are suggested standardised procedures, present results reinforce the need to uniform the procedure not only in the studies that diagnose these conditions but also in studies which present normative data. Further studies should evaluate which factors contribute to higher HGS values. Meanwhile, we suggest the adoption of the most recent ASHT protocol. In our opinion, this is the most detailed one and, thus, it is less probable to generate differences in HGS values between the studies. Nevertheless, we embrace that the complexity of this protocol may increase the difficulty in its application, especially in clinical practice. Future studies of these issues should include a complete description of the procedure, mentioning the deviations to the protocol.

## Abbreviations

ASHT: American Society of Hand Therapists
CHS: Cardiovascular Health Study
EWGSOP: European Working Group on Sarcopenia in Older People
HGS: Handgrip strength
IWGS: International Working Group on Sarcopenia
PRISMA: Preferred Reporting Items for Systematic Reviews and Meta-Analyses
